# SDC-YOLOv8: An Improved Algorithm for Road Defect Detection Through Attention-Enhanced Feature Learning and Adaptive Feature Reconstruction

**DOI:** 10.3390/s26020609

**Published:** 2026-01-16

**Authors:** Hao Yang, Yulong Song, Yue Liang, Enhao Tang, Danyang Cao

**Affiliations:** 1 School of Computer and Artificial Intelligence, Beijing Technology and Business University, Beijing 100048, China; pikx258@163.com (H.Y.); sungyulung@163.com (Y.S.); 13717521965@163.com (E.T.); 2 School of Artificial Intelligence and Computer, North China University of Technology, Beijing 100144, China; a23814022962025@163.com

**Keywords:** road defect detection, YOLOv8, attention mechanism, feature learning, feature reconstruction, real-time detection

## Abstract

Road defect detection is essential for timely road damage repair and traffic safety assurance. However, existing object detection algorithms suffer from insufficient accuracy in detecting small road surface defects and are prone to missed detections and false alarms under complex lighting and background conditions. To address these challenges, this study proposes SDC-YOLOv8, an improved YOLOv8-based algorithm for road defect detection that employs attention-enhanced feature learning and adaptive feature reconstruction. The model incorporates three key innovations: (1) an SPPF-LSKA module that integrates Fast Spatial Pyramid Pooling with Large Separable Kernel Attention to enhance multi-scale feature representation and irregular defect modeling capabilities; (2) DySample dynamic upsampling that replaces conventional interpolation methods for adaptive feature reconstruction with reduced computational cost; and (3) a Coordinate Attention module strategically inserted to improve spatial localization accuracy under complex conditions. Comprehensive experiments on a public pothole dataset demonstrate that SDC-YOLOv8 achieves 78.0% mAP@0.5, 81.0% Precision, and 70.7% Recall while maintaining real-time performance at 85 FPS. Compared to the baseline YOLOv8n model, the proposed method improves mAP@0.5 by 2.0 percentage points, Precision by 3.3 percentage points, and Recall by 1.8 percentage points, yielding an F1 score of 75.5%. These results demonstrate that SDC-YOLOv8 effectively enhances small-target detection accuracy while preserving real-time processing capability, offering a practical and efficient solution for intelligent road defect detection applications.

## 1. Introduction

Road surface defects, particularly potholes, pose significant threats to driving safety, accelerate vehicle structural wear, and substantially increase road maintenance costs [1,2,3]. In high-speed or complex traffic environments, potholes can cause vehicle loss of control, tire blowouts, and serious traffic accidents. Timely and accurate detection and repair of such defects are therefore essential for ensuring public safety and extending pavement service life [1,4]. However, traditional road inspection methods rely heavily on manual on-site surveys or vehicle-mounted inspections, which are labor-intensive, inefficient, and highly subjective. These limitations make conventional approaches unable to meet the large-scale, real-time, and fine-grained condition monitoring demanded by modern intelligent transportation systems [2,5].

With the rapid development of computer vision and deep learning, automated road defect detection based on image analysis has become an active research topic [1,5]. Convolutional neural networks (CNNs), with strong feature extraction and classification capabilities, have enabled automatic identification of potholes, cracks, and other surface defects from images or video streams [3,4]. Earlier work largely relied on traditional image processing techniques such as edge detection, texture analysis, and threshold segmentation. Although these methods can identify defects under controlled conditions, their performance is highly sensitive to imaging quality and environmental conditions. They struggle with variations in illumination, shadows, and background clutter, and have difficulty generalizing across defects with diverse shapes and scales [1,2].

The emergence of deep learning–based object detectors has substantially improved the accuracy and robustness of road defect detection. In particular, the YOLO (You Only Look Once) family of single-stage detectors has been widely adopted for pavement damage detection due to its end-to-end training, high detection speed, and competitive accuracy [3,6]. YOLOv3 introduces multi-scale prediction to improve detection performance while retaining real-time inference, but its ability to detect small objects remains limited [7]. YOLOv5 incorporates enhanced feature fusion and a lightweight design to better balance accuracy, speed, and deployment convenience [8,9]. YOLOv6 further optimizes inference speed and parameter efficiency for industrial scenarios, but still suffers from missed detections and false alarms under complex road conditions [10]. As the latest in the series, YOLOv8 upgrades the backbone, feature fusion structure, and anchor-free detection head, improving overall accuracy and generalization across multi-class defect detection tasks [6,11].

To cope with the specific challenges of road defect detection, numerous improvements to YOLO-series models have been proposed. Feature pyramid structures such as FPN, PAN, and BiFPN have been introduced to strengthen multi-scale feature fusion and improve the detection of defects with different sizes [3,9,10]. Attention mechanisms, including coordinate attention, channel attention, and spatial attention, have been embedded into YOLO backbones or necks to enhance feature representation and localization of defect regions [6,8,11]. In addition, methods that exploit dynamic feature enhancement, multi-source data fusion, and domain-specific cues (e.g., road surface texture, ground-penetrating radar subsurface signals) have been explored to improve robustness under challenging conditions such as illumination changes, background texture similarity, and partial occlusion [1,7,8].

Despite these advances, YOLOv8 still faces three prominent challenges in pothole detection. First, potholes are typical small objects with irregular sizes, shapes, and blurred edges. In low-resolution images or distant views, their features are weak and easily confused with the background, leading to insufficient feature representation and loss of contextual semantics, which directly reduces detection accuracy [12,13,14,15,16]. Second, complex road environments—characterized by shadows, illumination variation, pavement-texture similarity, water accumulation, and dirt occlusion—further increase the risk of missed detections and false alarms [13,15]. Such interference degrades feature extraction and weakens generalization across different scenes. Third, practical deployment on mobile or in-vehicle embedded devices requires not only high accuracy but also lightweight models and strict real-time performance [12,16]. Many high-accuracy detectors rely on deep backbones and heavy enhancement modules, resulting in high computational cost and inference latency that are unsuitable for resource-constrained platforms.

In practice, YOLOv8-based detectors for road damage tend to exhibit several typical failure modes: (i) missed detections of very small or distant potholes with blurred boundaries and weak texture contrast; (ii) false positives caused by specular reflections on wet asphalt, shallow water puddles, or tar patches that mimic pothole appearance; (iii) unstable localization of irregular, elongated defects whose contours are only partially visible; and (iv) degraded performance under low-contrast conditions such as fog, overcast weather, or strong shadows. These issues suggest that the baseline YOLOv8n configuration may lack sufficiently rich multi-scale context, suffer from aliasing in the upsampling path, and fail to exploit direction-aware positional cues to disambiguate defects from background clutter.

Beyond the above advances, several recent studies have proposed YOLO-based detectors specifically tailored for pavement or industrial defect inspection. For example, Maeda et al. constructed one of the earliest public road damage datasets using smartphone images and benchmarked several one-stage detectors such as SSD and YOLO, showing that CNN-based detectors can already achieve practical accuracy for crack and pothole detection in real-world scenes [17]. Building on YOLOv5, Sami et al. designed an improved pavement damage detector by integrating Efficient Channel Attention, label smoothing, K-means++ anchor generation, Focal Loss and an extra prediction layer, and reported higher mAP and F1-score on the RDD2022 dataset compared with the YOLOv5s and YOLOv8s baselines [18]. Wan et al. further introduced YOLO-LRDD, a lightweight YOLOv5s-based network that adopts depthwise separable convolutions and an enhanced feature pyramid to reduce parameters while maintaining competitive accuracy for embedded road damage detection [19]. On top of YOLOv8, several variants such as YOLOv8-PD, OBC-YOLOv8 and YOLO-DCNet modify the backbone or neck with deformable convolutions, attention modules and scale-aware feature fusion to better handle small cracks, complex textures and illumination variations on pavement surfaces [20,21]. In parallel, segmentation-based architectures (e.g., PDSNet) have also been explored to obtain pixel-level damage masks instead of bounding boxes [22], but they usually suffer from higher computational cost and lower inference speed, which limits their deployment in real-time inspection scenarios. Beyond road surfaces, YOLO-based detectors have also been widely adopted in industrial defect inspection, where targets are typically small, low-contrast, and embedded in complex textures. For example, Mao et al. [23] developed a YOLO-based framework for automated defect detection in mass-produced electronic components under strict real-time constraints. Their findings highlight that, even in controlled factory environments, small-defect detection remains highly sensitive to feature representation quality, illumination variation, and computational budgets—challenges that are closely aligned with those in road-pothole inspection. Our work can be viewed as extending these insights from industrial production lines to on-road infrastructure, with a particular focus on lightweight architectural refinements suitable for edge deployment. Very recently, YOLOv9 has been proposed as a new benchmark in the YOLO family [24], introducing programmable gradient information (PGI) and lightweight task-aligned features to improve the convergence and accuracy–efficiency trade-off on large-scale detection benchmarks. Compared with YOLOv8, YOLOv9 primarily targets general-purpose multi-class detection on datasets such as COCO, and its reference implementations are optimized for high-end GPUs. While these advances are highly relevant, our focus in this work is on single-class road-defect detection under edge-compute constraints, where the overall system budget (FLOPs, latency, memory) and module compatibility with resource-limited platforms are key considerations. Incorporating PGI or YOLOv9-style task-aligned features into SDC-YOLOv8 is an interesting direction for future research.

Existing YOLO-based road and industrial defect detectors alleviate some of these issues but do not fully resolve them in our setting. Many methods focus on channel-wise attention or generic backbone scaling, which improves global feature expressiveness but leaves small, irregular defects under complex illumination only partially addressed. Others adopt heavier backbones or multi-branch feature pyramids that improve accuracy at the cost of latency and memory, making them less suitable for edge deployment on in-vehicle platforms. Consequently, there remains a gap for a lightweight yet error-pattern–aware design that explicitly targets small, low-contrast, reflection-contaminated, and irregular potholes while preserving real-time performance.

To address these issues, this paper proposes SDC-YOLOv8, an improved road surface defect detection model built on the YOLOv8 framework that combines attention-enhanced feature learning with adaptive feature reconstruction. SDC-YOLOv8 introduces three lightweight components deployed at different stages of the detector. First, an SPPF-LSKA module is integrated into the backbone by combining Spatial Pyramid Pooling-Fast (SPPF) with Large Separable Kernel Attention (LSKA), thereby enriching deep multi-scale context and modeling irregular, low-contrast potholes while maintaining computational efficiency. Second, a DySample dynamic upsampling module is embedded in the neck to replace conventional fixed interpolation; by learning sampling-point offsets, DySample performs sampling-based adaptive feature reconstruction, better preserving fine-scale cues and mitigating aliasing in weak-texture regions. Third, Coordinate Attention (CA) modules are inserted between the neck and the detection heads to separately model spatial information along the height and width dimensions via directional encoding, enhancing localization and recognition performance under complex conditions such as illumination variation, texture similarity, and partial occlusion.

The main contributions of this work are threefold. (1) We propose an error-pattern–driven architecture refinement for YOLOv8n. By analyzing typical failure modes in pothole detection—missed small and low-contrast defects, reflection-induced false positives, and unstable localization of irregular targets—we design a three-stage refinement strategy that, respectively, targets backbone-level context enhancement, neck-level feature reconstruction, and head-level localization. (2) We develop a lightweight SDC-YOLOv8 design that balances accuracy and deployability. By integrating SPPF-LSKA, DySample, and Coordinate Attention at carefully chosen locations within YOLOv8n, the proposed model achieves a favorable Pareto trade-off: compared with the YOLOv8n baseline, SDC-YOLOv8 improves mAP@0.5 by 2.0 percentage points and Recall by 1.8 percentage points, with only about +0.16 M parameters and +0.6 GFLOPs overhead at 640 × 640 resolution, while still running at 85 FPS on a single TITAN X GPU. (3) We provide comprehensive empirical analysis and engineering insights for road defect detection under complex conditions. Beyond headline metrics, we conduct an in-depth ablation study, decompose latency and FLOPs, and present qualitative failure analyses in challenging scenes with reflections, water accumulation, low contrast, and densely distributed small defects. These results offer practical guidelines for selecting and combining lightweight modules in resource-constrained defect detection systems and demonstrate that the proposed staged design yields mutually reinforcing gains rather than merely stacking isolated components.

## 2. Materials and Methods

### 2.1. Dataset and Annotation

We employed the public pothole dataset available on Kaggle as the basis of this study [25]. The dataset contains real-world road images with potholes of diverse shapes and scales on two common pavement materials: asphalt and concrete. To ensure representativeness and data quality, we first conducted a strict screening and cleaning procedure. Duplicate frames, severely blurred or overexposed images, and samples with missing or clearly inconsistent annotations were removed or corrected. This process reduced label noise and improved the reliability of subsequent training and evaluation.

All pothole instances were then manually annotated as a single category (“pothole”). Initial labeling was performed using a general-purpose image annotation tool, followed by a thorough consistency review by the research team. For challenging cases such as occlusions, overlapping defects, and strong shadows, we followed a unified guideline: the bounding box should cover the visible contour of the pothole as completely as possible while avoiding excessive background. This criterion helped maintain consistent box shapes and boundaries across the dataset and improved the comparability of experimental results. The final annotations were converted to and validated in YOLO format (.txt) and paired one-to-one with their corresponding image files to support reproducible data loading and training.

To approximate typical in-vehicle or edge deployment conditions, all input images were resized to a fixed resolution of 640 × 640 pixels. Before training, we applied geometric and photometric normalization, including proportional resizing, center cropping when necessary, and pixel-intensity normalization, to reduce variation caused by different cameras, shooting distances, and viewing angles. Considering the long-tail distribution of pothole sizes and viewpoints in real road environments, we adopted moderate data augmentation during training, such as random horizontal flipping, mild color jittering, and small-scale perturbations. All augmentation operations were disabled during evaluation, so that test results remained comparable and reproducible.

In total, the curated dataset consists of 1100 images, randomly split in an 8:2 ratio into 880 training images and 220 test images. A fixed random seed was used when generating this split to keep the correspondence between images and labels stable across runs and to mitigate distribution drift between experiments. The main text reports results under this two-part train/test setting for both baseline and comparative experiments. If required, we can also adopt a three-part split (training/validation/testing) without altering the main conclusions.

**Figure 1:**
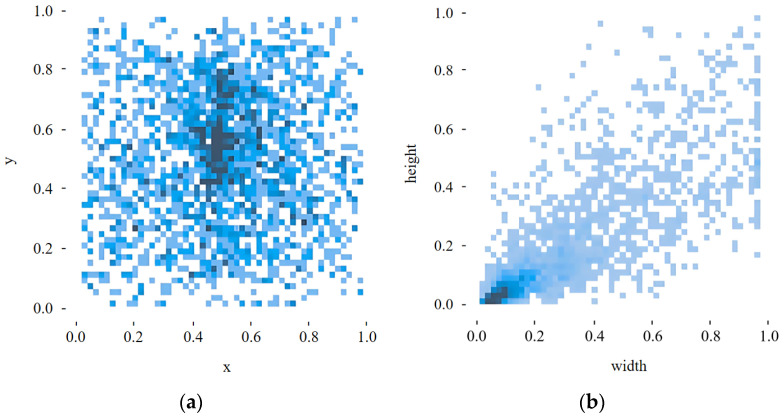
(**a**) Distribution of the x dimension in the dataset; (**b**) Distribution of the width dimension in the dataset. Data set analysis.

To better understand the data distribution, we performed a statistical analysis of the annotations, as illustrated in Figure 1. Figure 1a shows a heatmap of bounding-box centers, where darker regions indicate higher target density and reveal spatial “hot zones” and typical viewpoints across scenes. Figure 1b presents the joint distribution of object width and height under the normalized 640 × 640 resolution, highlighting the long-tail characteristics and aspect-ratio variability of potholes. These plots confirm that the dataset contains both large, clear potholes and small, weak-texture or partially occluded instances. They also show that shadows, reflections, and pavement-like textures occur frequently, providing challenging samples for robustness evaluation.

Overall, the constructed dataset exhibits substantial diversity in pavement material, defect scale, and scene conditions. The standardized pipeline of cleaning, annotation, validation, and visualization ensures that the data used in this work are of high quality and that all subsequent experiments—including ablation and comparative studies—are carried out on a consistent and auditable basis.

In terms of scene coverage, the curated dataset spans a variety of road types, including dense city-center streets, suburban arterials, and lower-traffic rural segments. Although the original Kaggle annotations do not explicitly label “city center” versus “suburban” locations, visual inspection confirms that both highly cluttered urban environments (with buildings, lane markings, parked vehicles, and traffic signs) and more open road scenes are represented. As a result, the proposed SDC-YOLOv8 detector is intended for deployment in both city-center and suburban settings, where the timely detection of small potholes is particularly critical for driving safety and pavement maintenance planning.

Although the curated dataset comprises 1100 images, which is modest compared with large-scale benchmarks, it covers a wide variety of pavement materials, viewpoints, and challenging conditions (reflections, shadows, low contrast). For a single-class detection task with standardized preprocessing and augmentation, our experiments indicate that the dataset is sufficient to support stable training and reproducible comparisons across YOLO variants and module configurations.

### 2.2. Experimental Environment

We adopt PyTorch (version: 2.0.1, manufactured by Meta Platforms, Inc.) as the primary deep learning framework. Built on Python and offering mature tensor computation with GPU acceleration, PyTorch provides a stable operator ecosystem and integrates well with mainstream inference backends, making it suitable for both research and deployment-oriented object detection. All experiments were conducted on Ubuntu 18.04 LTS with 16 GB of host memory, an Intel Xeon E5-2683 v3 CPU, and a single NVIDIA GeForce GTX TITAN X GPU, NVIDIA Corporation, Santa Clara, CA, USA with 8 GB of VRAM. The programming language was Python 3.8, and the CUDA toolkit version was 11.3, which is compatible with the employed PyTorch/torchvision stack and sufficient to fully utilize the available GPU resources on an 8 GB card.

To ensure fair comparison and reproducibility, all training and inference experiments were performed on this unified software/hardware stack. Unless otherwise specified, all models shared the same data preprocessing and inference protocol. Training was carried out in FP32 precision. Data loading used four worker threads to mitigate I/O bottlenecks. Random seeds were fixed at startup (for Python, NumPy, and PyTorch), and appropriate cuDNN settings (enabling cudnn.benchmark while keeping cudnn.deterministic consistent where necessary) were selected to balance runtime performance and determinism. During evaluation, we adopted single-scale inference at 640 × 640 pixels with common confidence and NMS thresholds, ensuring that all detectors were assessed under identical conditions.

Table 1 summarizes the training hyperparameters. We set the number of epochs to 200 to cover both the convergence phase and a stable fine-tuning phase. The batch size was fixed at 8, reflecting the memory constraints of an 8 GB GPU while maintaining reasonable throughput and stable batch statistics. Stochastic gradient descent (SGD) with momentum 0.937 and an initial learning rate $lr_{0}=0.01$ was used, a configuration widely validated for YOLO-series and other single-stage detectors, and offering a good trade-off between convergence speed and final accuracy. The input resolution imgsz=640 represents a practical compromise for road-defect detection: it preserves small-object discernibility while remaining compatible with real-time, edge-oriented deployment. For data augmentation, we follow standard YOLO practice. Mosaic augmentation is enabled in the early training stage to enrich distributional diversity and boost recall, and then disabled in the final 10 epochs (close_mosaic = 10) so that the model fine-tunes on a distribution closer to the inference setting. Default color jitter and horizontal flipping are applied when they do not change the defect semantics, thereby improving robustness. The number of data-loading workers (workers = 4) is chosen to increase input throughput during training and reduce GPU idle time.

For the evaluation protocol, the train/test split described in Section 2.1 was kept fixed for all runs to avoid distribution drift and to support consistent comparative conclusions. After training, we report unified metrics on the test set, including Precision, Recall, and mAP@0.5, as well as engineering-oriented indicators such as FLOPs and a decomposition of end-to-end (E2E) latency into preprocessing, forward pass, and post-processing (as detailed in the corresponding tables and Section 3 and Section 4). To reduce the influence of single-run stochasticity, key comparisons were repeated and variance was inspected. If required by the venue, we can additionally provide bootstrap confidence intervals to quantify uncertainty in metrics such as mAP.

We deliberately chose stochastic gradient descent (SGD) with momentum, rather than more recent adaptive optimizers such as Adam or AdamW, for two reasons. First, SGD with momentum is the default and most widely validated configuration in the YOLO family, and has consistently yielded strong performance for single-stage object detectors under comparable settings. Second, on our relatively small, single-class pothole dataset, preliminary experiments with AdamW under the same training schedule led to slightly faster initial loss reduction but noticeably stronger overfitting and less stable validation curves near convergence. By contrast, SGD with momentum provided a better trade-off between final test mAP and training stability. For these reasons, we adopted SGD as the primary optimizer in all reported experiments.

Overall, our design principle is to operate within the compute and memory envelope typical of edge or vehicle-mounted platforms while enforcing a unified train–inference protocol and reproducible implementation details. This yields a stable, auditable foundation for subsequent baseline comparisons, ablation experiments, and engineering trade-off analyses (speed/size/latency versus accuracy). The same environment is used throughout Section 3 for all comparative and qualitative experiments, ensuring that conclusions are drawn from a consistent pipeline.

### 2.3. Evaluation Metrics

To ensure fair and reproducible comparison, all detectors are evaluated under a unified protocol: single-scale inputs of 640 × 640 pixels, batch size = 1, fixed confidence and NMS thresholds, and the hardware/software stack described in Section 2.2. Detection correctness is determined by matching predicted boxes to ground truth using the Intersection over Union (IoU) criterion in Equation (1). For a given IoU threshold τ, a prediction is regarded as a true positive (TP) if its category is correct and its IoU with a ground-truth box is at least τ; otherwise, it is counted as a false positive (FP), and any unmatched ground-truth box is treated as a false negative (FN).

At a fixed score threshold, Precision and Recall measure false-alarm control and missed-detection control, respectively, and are computed from TP, FP, and FN as in Equations (2) and (3). Sorting predictions by confidence and accumulating TP/FP yields the precision–recall (PR) curve over the recall range [0, 1]. For a single class at the IoU threshold τ, the Average Precision (AP) is defined as the area under the interpolated PR curve (Equation (4)). For multi-class detection, the Mean Average Precision (mAP) is the arithmetic mean of per-class AP, following Equation (5). Since our task involves a single class (“pothole”), mAP@0.5 is numerically identical to AP@0.5. When reporting mAP@0.5:0.95, we follow the COCO protocol and average AP over τ∈{0.50,0.55,…,0.95}, consistent with Equation (5).

Model complexity and memory footprint are evaluated from both theoretical and implementation perspectives. The floating-point operations required for a single 640 × 640 forward pass are reported as GFLOPs and computed according to Equation (6), providing a device-agnostic proxy for computational cost. The total number of learnable parameters (Params) and the serialized checkpoint size (Model Size) characterize the memory demand. Under FP32 storage, Model Size grows approximately linearly with Params as expressed in Equation (7), with minor deviations due to auxiliary buffers and serialization overhead.

Runtime behavior on the target platform is quantified by end-to-end (E2E) latency per image, measured in milliseconds and decomposed additively into preprocessing, forward inference, and post-processing, as in Equation (8). Here, Pre includes input decoding, geometric resizing, intensity normalization, and host-to-device transfer; Fwd denotes the GPU forward pass, which typically dominates the latency budget; and NMS encompasses candidate decoding and non-maximum suppression, whose cost increases with proposal density. Throughput (images per second) and latency are approximately inversely related, as summarized in Equation (9), with small discrepancies arising from pipeline overlap and I/O jitter.

All measurements are obtained under consistent settings: single-scale 640 × 640 inputs, batch size = 1, FP32 inference, shared confidence/NMS thresholds across models, fixed random seeds during training to reduce variance, and repeated timing after warm-up to stabilize wall-clock estimates. Under these conventions, the quantities reported in Table 2—mAP@0.5, mAP@0.5:0.95, GFLOPs, Params, Model Size, and E2E latency with its components—are directly comparable across detectors and reproducible on the declared experimental platform.

To make the reported numbers more reproducible, we additionally examined training stability and run-to-run variability. For the main configurations (YOLOv8n and SDC-YOLOv8), we trained the models multiple times with different random seeds while keeping all hyperparameters and the train/test split fixed. The resulting learning curves did not exhibit divergence or oscillatory behavior, and the final test metrics varied only slightly across runs. For transparency, we provide the per-epoch training and validation curves, together with per-run summaries of Precision, Recall and mAP@0.5, so that readers can directly inspect variance and, if desired, compute confidence intervals for their own analyses. Concretely, for YOLOv8n and SDC-YOLOv8, we repeated training with three random seeds and observed that the standard deviation of mAP@0.5 across runs was below 0.8 percentage points. Detailed per-epoch curves and per-run summaries are provided, enabling readers to assess run-to-run variability.

In addition to Precision, Recall, and mAP, we also report the F1 score, defined as the harmonic mean of Precision and Recall, as in Equation (10).

Unless otherwise noted, all metrics in the result tables are computed on the held-out test set described in Section 2.1, using the unified evaluation protocol of Section 2.3.

(1) $$\mathrm{IoU}(B_{p},B_{g})=\frac{\left|B_{p}\cap B_{g}\right|}{\left|B_{p}\cup B_{g}\right|}$$

(2) $$\mathrm{Precision}=\frac{TP}{TP+FP}$$

(3) $$\mathrm{Recall}=\frac{TP}{TP+FN}$$

(4) $$\mathrm{AP}=\int_{0}^{1}P_{\mathrm{interp}}(R)dR_{P_{\mathrm{interp}}(R)\;=\;\max_{\tilde{R}\geq R}P(\tilde{R})}$$

(5) $$\mathrm{mAP}=\frac{1}{C}\sum_{c=1}^{C}{\mathrm{AP}}_{c}$$

(6) $$\mathrm{GFLOPs}=\frac{\mathrm{ops}\;\mathrm{per}\;\mathrm{image}}{10^{9}}.$$

(7) $$\mathrm{Model}\;\mathrm{Size}\;(\mathrm{MB})\approx \frac{\mathrm{Params}\times 4\;\mathrm{bytes}}{1024^{2}}$$

(8) E2E=Pre+Fwd+NMS.

(9) $$\mathrm{FPS}\approx \frac{1000}{E2E\;(\mathrm{ms})}$$

F_1_ = 2·Precision·Recall/(Precision + Recall) (10)

### 2.4. Baseline Algorithm: YOLOv8

**Figure 2:**
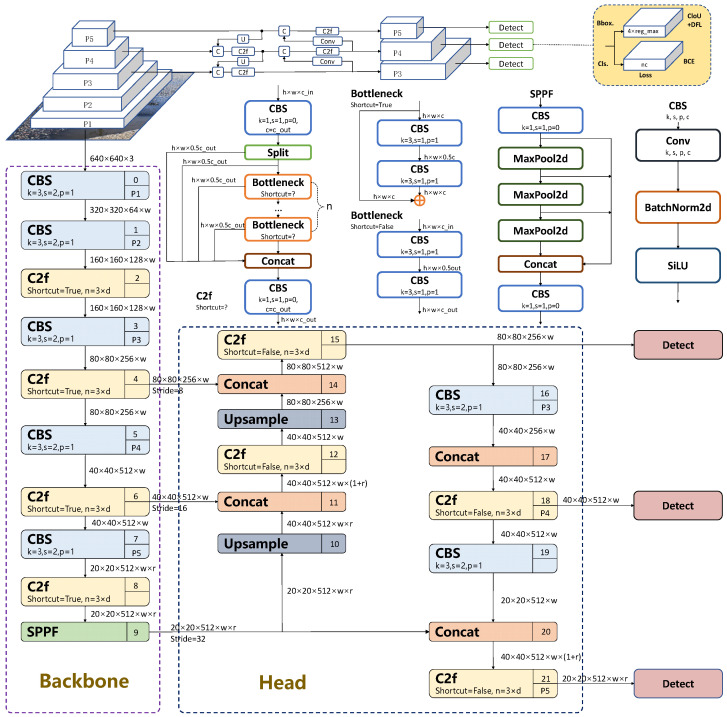
YOLOv8 network structure diagram.

YOLOv8 is a state-of-the-art one-stage detector family with multiple scaled variants (n/s/m/l/x) [25,26]. In this work, we use YOLOv8n as the baseline due to its favorable accuracy–efficiency trade-off for edge/vehicle-mounted road inspection. This unified framework supports training for object detection, instance segmentation, and image classification—surpassing its predecessors in both detection speed and accuracy. This paper focuses on its application in object detection; its network structure is divided into four parts: input end, backbone network, fusion layer, and detection head (Figure 2).

The Mosaic data-augmentation strategy, originally popularized in YOLOX, is also adopted in YOLOv8. Mosaic is enabled in the early training stage to increase sample diversity and improve robustness, and then disabled in the final 10 epochs so that the network can fine-tune on images whose distribution is closer to the inference setting [18]. In addition, an adaptive image-scaling strategy resizes the original images to a fixed input resolution, which simplifies the detection pipeline and helps improve accuracy.

The backbone network consists of CBS, C2f, and SPPF modules. The CBS block is composed of a convolution layer (Conv), batch normalization (BN), and the SiLU activation function, and is responsible for basic feature transformation. The newly introduced C2f module in the YOLO series acts as the main feature extractor, enhancing gradient flow and feature reuse. The Spatial Pyramid Pooling Fast (SPPF) module aggregates information from multiple receptive fields, enabling the backbone to better capture objects with diverse scales.

The neck adopts an FPN–PAN structure. The Feature Pyramid Network (FPN) propagates high-level semantic information in a top–down manner, while the Path Aggregation Network (PAN), as an extension of FPN [27,28] introduces bottom–up paths and lateral connections to strengthen localization cues. This bidirectional design facilitates effective fusion of low-level and high-level feature maps.

The detection head uses an anchor-free assignment strategy, which removes the need for predefined anchor boxes. It directly regresses the center coordinates, width, and height of targets on feature maps at different scales, thereby reducing computational overhead and simplifying optimization. By exploiting multi-scale feature maps from the neck, the detector can accurately classify and localize large, medium, and small potholes.

### 2.5. Improved Algorithm: SDC-YOLOv8

**Figure 3:**
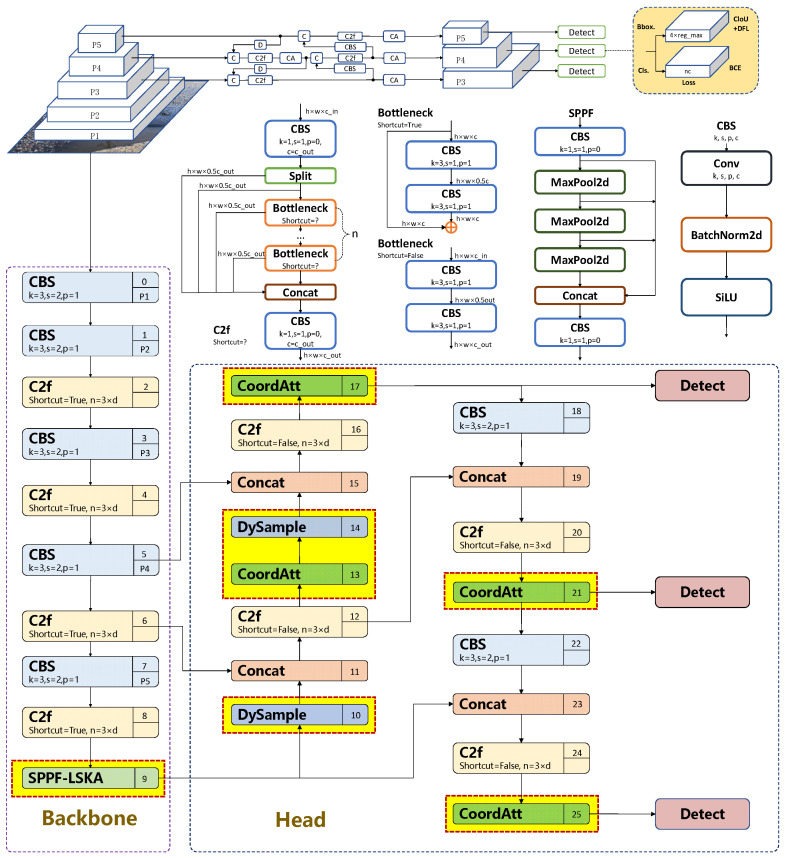
Overall architecture of the proposed SDC-YOLOv8 detector and the insertion points of SPPF-LSKA, DySample, and Coordinate Attention.

At a high level, the proposed SDC-YOLOv8 detector retains the original YOLOv8n backbone–neck–head topology, and only modifies a few key blocks with lightweight modules at clearly defined locations (Figure 3). The overall design follows a top–down refinement strategy, from deep context modeling in the backbone, through feature reconstruction in the neck, to attention-enhanced localization in the head.

First, in the backbone, the original SPPF block at the deepest stage (P5) is replaced by the SPPF-LSKA module (yellow box at index 9 in Figure 3), which augments multi-scale context aggregation with large-kernel attention while preserving the input–output tensor shapes. Second, in the neck, two DySample units (yellow boxes at indices 10 and 14) are inserted along the upsampling paths from P5 to P4 and from P4 to P3, substituting the default nearest-neighbor upsampling and enabling content-adaptive feature reconstruction. Third, at the interface between the neck and the detection head, Coordinate Attention (CoordAtt) blocks (yellow/green boxes at indices 13, 17, 21, and 25) are attached to the P3, P4, and P5 feature maps to enhance direction-aware and position-aware localization before prediction.

These three modifications leave the stride configuration, detection heads, and training/inference pipeline of YOLOv8n unchanged, while assigning complementary roles to each added component: deep context modeling (SPPF-LSKA) in the backbone, neck-level feature refinement (DySample), and head-level attention (CoordAtt) within a unified architecture.

Prior to experimentation, each iteration of the YOLOv8 model undergoes rigorous testing, with YOLOv8n emerging as the benchmark model in this paper based on a thorough analysis of the experimental results. While larger YOLOv8 variants (e.g., YOLOv8s) may offer marginal accuracy gains, road pothole detection is dominated by small targets and complex backgrounds, for which scaling the network provides limited benefit relative to the increased computational cost. Extracting precise feature information for potholes in such scenarios remains challenging, often leading to false detections and missed targets. Therefore, this paper introduces an enhanced road pothole detection model based on YOLOv8n, with its network architecture depicted in. The end-to-end workflow of SDC-YOLOv8 from input image to final detections is summarized in Algorithm 1.
**Algorithm 1:** Inference pipeline of SDC-YOLOv8Input: RGB road image I Output: final pothole detections D 1: $I^{\prime }$ ← resize and normalize I to 640×640 2: //backbone with SPPF-LSKA at stage P5 3: $F_{P5}$ ← Backbone_YOLOv8n_SPPF_LSKA ($I^{\prime }$) 4: //neck with DySample and FPN–PAN fusion 5: $\left(F_{P3},F_{P4},F_{P5}\right)$ ← Neck_FPNPAN_DySample ($F_{P5}$) 6: //apply Coordinate Attention on each scale 7: for each scale s∈{P3,P4,P5} do 8: $F_{s}^{\prime }$ ← CoordinateAttention ($F_{s}$) 9: end for 10: //anchor-free detection heads 11: Y ← DetectHeads ($F_{P3}^{\prime },F_{P4}^{\prime },F_{P5}^{\prime }$) 12: //confidence filtering and NMS 13: $Y_{\mathrm{conf}}$ ← {$y\in Y|y.\mathrm{score}\geq \tau_{\mathrm{conf}}$} 14: D ← NMS ($Y_{\mathrm{conf}},\tau_{\mathrm{nms}}$) 15: return D

#### 2.5.1. SPPF-LSKA Module Design

In conventional convolutional neural networks, the architecture is usually designed for a fixed input resolution. When images must be cropped, flipped, or rescaled to fit this resolution, aspect-ratio distortion and loss of contextual information can degrade detection accuracy. To relax this constraint, He et al. proposed Spatial Pyramid Pooling (SPP), which enables the network to accept inputs of varying sizes. SPP applies pooling with multiple bin sizes on the final convolutional feature map and concatenates the results into a fixed-length feature vector, thereby aggregating multi-scale responses while preserving information from each scale.

On this basis, the Spatial Pyramid Pooling-Fast (SPPF) variant was developed. SPPF replaces the parallel multi-kernel pooling of SPP with a sequence of pooling layers using a single kernel size, which effectively mimics multi-scale receptive fields. This serial design reduces computational cost and memory usage while maintaining the ability to capture rich multi-scale context, making it more suitable for real-time detectors such as YOLOv8.

To further enhance contextual modeling with low overhead, we employ the Large Separable Kernel Attention (LSKA) module. LSKA factorizes large two-dimensional convolution kernels in the attention branch into cascaded one-dimensional convolutions along the horizontal and vertical directions. This separable formulation allows very large effective kernels to be introduced into the attention mechanism without additional heavy blocks, providing a more efficient alternative to standard LKA and enabling stronger long-range dependency modeling in the backbone.

**Figure 4:**
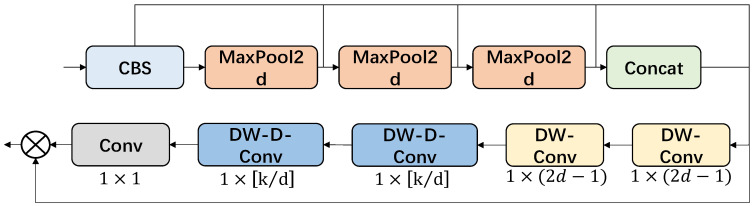
Architecture of the proposed SPPF-LSKA module. “MaxPool2d” denotes 5 × 5 max-pooling with stride 1, so all pooled feature maps preserve the same spatial resolution as the input. The original and pooled feature maps are concatenated along the channel dimension and then processed by depthwise and dilated convolutions to capture multi-scale contextual information.

In our implementation, following the SPPF design of YOLOv5/YOLOv8, all max-pooling layers in the SPPF-LSKA block adopt a kernel size of 5 × 5 with stride 1. Let $X\in R^{C\times H\times W}$ denote the input feature map. Three consecutive max-pooling operations generate $P_{1},\;P_{2},$ and $P_{3}$, each having the same spatial resolution H×W as X. Since no spatial down-sampling is introduced (stride = 1), the four feature maps $\left[X,P_{1},P_{2},P_{3}\right]$ can be safely concatenated along the channel dimension without any additional resizing, effectively mimicking multi-scale receptive fields while preserving the original spatial size. The architecture of the proposed SPPF-LSKA module is illustrated in Figure 4.

Drawing inspiration from the aforementioned techniques, this paper introduces LSKA into the SPPF module, culminating in the SPPF-LSKA module. The structure of this innovative module is depicted in the output of the LSKA part is shown below:

(11) $${\bar{Z}}^{C}=\sum_{H,W}W_{(2d-1)\times 1}^{C}*\left(\sum_{H,W}W_{1\times (2d-1)}^{C}*F^{C}\right)$$

(12) $$A^{C}=W_{1\times 1}*Z^{C}$$

(13) $${\bar{F}}^{C}=A^{C}⊗F^{C}$$

Here, ∗ signifies the convolution operation, while ⨂ represents the Hadamard product. The notation $Z^{C}$ denotes the output of the depth convolution, which utilizes a kernel size of (2d − 1) × (2d − 1). This kernel effectively captures local spatial information, thereby compensating for any potential grid effect induced by subsequent depth convolution steps (as seen in Equation (13)).

The size of the depth convolution kernel is determined by (⌊k/d⌋ × ⌊k/d⌋), where ⌊·⌋ represents the floor function, rounding down to the nearest integer. The dilated depth convolution, on the other hand, is tasked with capturing the global spatial information that is generated as an output from the depth convolution.

Furthermore, the LSKA module employs a novel strategy of decomposing the 2D convolution kernels found in deep convolutional layers into a cascade of horizontal and vertical 1D kernels. This decomposition allows for a more efficient and targeted extraction of spatial features.

#### 2.5.2. DySample Module

To streamline model efficiency, the lightweight DySample module replaces the traditional Upsample component. DySample is an ultra-lightweight, high-performance dynamic upsampling technique—addressing limitations of kernel-based dynamic upsamplers (CARAFE [29], FADE [30], SAPA [31]) which have high computational overhead (due to time-intensive dynamic convolutions and additional subnets for dynamic kernel generation) and restricted applicability (FADE and SAPA rely on high-resolution feature responses).

**Figure 5:**
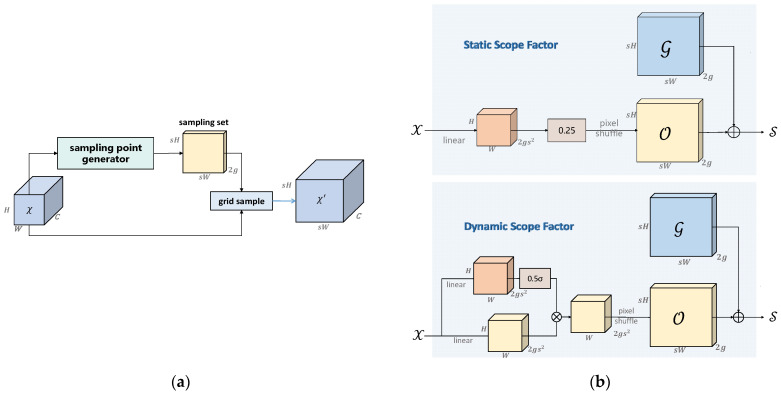
Sampling-based dynamic upsampling and module designs in DySample. (**a**) The sampling set is generated by the sampling point generator, with which the input feature is re-sampled by the grid sample function. In generator (**b**), the sampling set is the sum of the generated offset and the original grid position.

Proposed by Liu [32], DySample circumvents dynamic convolutions by formulating upsampling as a point sampling technique—saving resources and enabling straightforward implementation using standard PyTorch built-in functions (Figure 5).

#### 2.5.3. CA Mechanism

In the detection of road defects, two primary factors often hinder accurate recognition: image blur and the similarity between potholes and ground color. However, the introduction of the CA module has brought significant advancements. This module endows the model with deeper levels of direction awareness and location awareness information, thereby enhancing its ability to precisely locate and recognize defect target areas.

**Figure 6:**
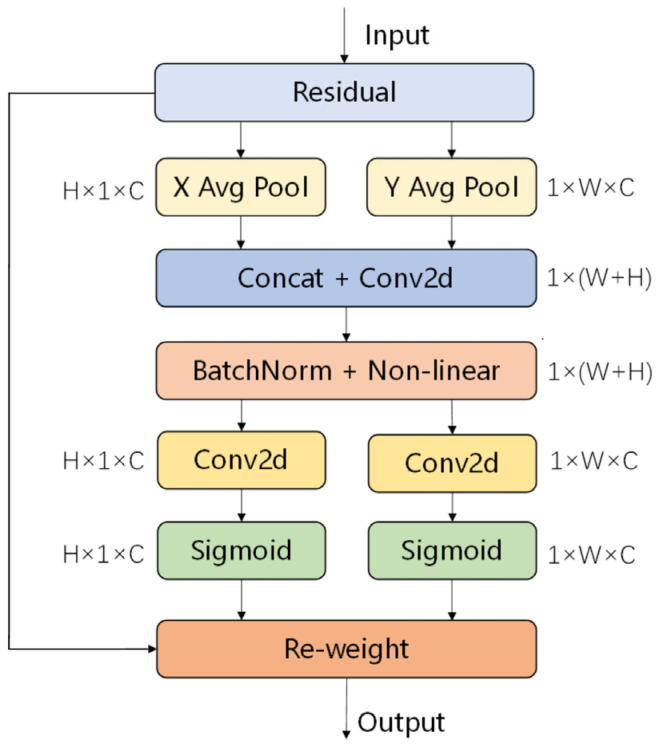
Coordinate Attention mechanism flow chart.

The CA mechanism module serves a crucial purpose: to enhance the mobile network’s learning feature expression capabilities. This methodology dissects the tensor X = [$x_{1}$, $x_{2}$, …, $x_{c}$] ∈ R^(H × W × C) and transforms it into an equally sized tensor Y = [$y_{1}$, $y_{2}$, …, $y_{c}$] ∈ R^(H × W × C). Figure 6 provides a detailed illustration of the implementation process of this CA mechanism.

To capture the attention to both the width and height dimensions of an image, and encode its precise location information, the CA module initially segregates the input feature map into two distinct orientations. It then proceeds to compute the global average independently for each orientation, resulting in feature maps that represent the image’s characteristics in both directions. The specific steps involved in this process are outlined in Equations (14) and (15).

(14) $$z_{c}^{h}\left(h\right)=\frac{1}{W}\sum_{0\leq i\leq W}x_{c}(h,i)$$

(15) $$z_{c}^{w}\left(w\right)=\frac{1}{H}\sum_{0\leq i\leq H}x_{c}(j,w)$$

Subsequently, the feature maps derived from the global receptive field, encompassing both the width and height dimensions, are seamlessly concatenated. This amalgamation of feature maps is then directed into a shared 1 × 1 convolutional module, where their dimensionality is efficiently reduced to C/r. Following batch normalization, the refined feature map F1 undergoes a Sigmoid activation function, ultimately yielding a feature map f with a distinct shape of 1 × (W + H) × C/r, as precisely defined in Equation (16).

(16) $$f=\delta (F_{1}([z^{h},z^{w}]))$$

Next, utilizing the original height and width as a reference, the feature map f undergoes convolution with a 1 × 1 convolutional kernel, resulting in feature maps $F_{h}$ and $F_{w}$ that maintain the identical dimensions as the original. Subsequently, after traversing the Sigmoid activation function, separate attention weights “$g^{h}$” and “$g^{w}$” are derived, specifically targeting the height and width dimensions of the feature map, respectively. This intricate process is illustrated in Equations (17) and (18).

(17) $$g^{h}=\sigma (F_{h}(f^{h}))$$

(18) $$g^{w}=\sigma (F_{w}(f^{w}))$$

Once the aforementioned calculations have been completed and the attention weights $g^{h}$ and $g^{w}$ have been determined, a multiplicative weighting mechanism is applied to the original image. This transformation leads to the generation of an enhanced image, along with its corresponding refined image feature values. This comprehensive process is concisely encapsulated in Equation (19).

(19) $$y_{c}(i,j)=x_{c}(i,j)\times g_{c}^{h}(i)\times g_{c}^{w}(j)$$

For ease of reference, Table 2 summarizes where each module is inserted, its main hyper-parameters, and its incremental cost in parameters and FLOPs relative to the YOLOv8-n baseline. This centralized view complements Figure 2, Figure 3 and Figure 4 and allows readers to assess the architectural changes and their overhead at a glance.

Table 2 summarizes the architectural modifications introduced by SDC-YOLOv8 and their impact on model complexity. For each module (SPPF-LSKA, DySample, and Coordinate Attention), we list its insertion stage in the network, key design settings, and the additional parameters (Δ Params) and GFLOPs (Δ GFLOPs) relative to the YOLOv8n baseline. As shown in Table 2, SPPF-LSKA slightly increases the parameter count and computation at the deepest backbone stage but strengthens multi-scale context and long-range dependency modeling. DySample adds only a small number of parameters to the neck while replacing fixed nearest-neighbor upsampling with content-adaptive sampling, which improves detail reconstruction. Coordinate Attention introduces negligible overhead before the three detection heads, as it operates on already downsampled feature maps, but injects direction-aware and position-aware attention that benefits localization. Overall, the combined overhead is modest (+0.16 M parameters and +0.6 GFLOPs at 640 × 640), so the SDC-YOLOv8 model remains lightweight and well-suited to edge and in-vehicle deployment. As summarized in Table 2, SPPF-LSKA, DySample and CA together increase the parameter count by only 0.16 M and the FLOPs by 0.6 at 640 × 640, leaving the overall model firmly in the lightweight regime.

## 3. Results

### 3.1. Baseline Algorithm Prior Experiments

To identify the most suitable YOLOv8 variant for road-pothole detection, we conducted a controlled baseline study comparing all official versions (YOLOv8-n/s/m/l/x) on our self-compiled pavement-defect dataset under a single, unified pipeline. The dataset split was fixed across all experiments (train/val/test = 8/1/1; see Section 2.1), and the same annotations and image pre-processing procedures were used in every run. All models were trained with an identical schedule and augmentation configuration: 200 epochs, SGD optimizer with momentum 0.937, initial learning rate $lr_{0}=0.01$, input resolution of 640 × 640, and Mosaic augmentation disabled during the final 10 epochs. Evaluation was performed using single-scale inference at 640 × 640, batch size 1, and FP32 precision. Confidence and NMS thresholds followed Ultralytics defaults and were kept fixed to avoid threshold-tuning bias. All experiments were executed on the same hardware/software environment (Ubuntu 18.04, Python 3.8, CUDA 11.3, PyTorch, NVIDIA GTX TITAN X with 8 GB VRAM, four data-loading workers), with random seeds fixed to reduce run-to-run variance.

For performance reporting, we adopted mAP@0.5 (defined in Equation (5)) as the primary accuracy indicator and characterized efficiency using both GFLOPs per 640 × 640 forward pass (as a device-agnostic measure of computational complexity) and end-to-end FPS (as a device-specific measure of throughput, including pre- and post-processing). This joint accuracy–efficiency perspective reflects the practical constraints of real-time road inspection, where latency budgets and limited compute resources are as critical as detection accuracy. All results in Table 2 were obtained under these controlled conditions, ensuring that performance differences can be attributed to the model variants themselves rather than to training or evaluation confounders.

As shown in Table 3, the larger YOLOv8 variants (s/m/l/x) provide only minor improvements in mAP@0.5 relative to YOLOv8-n, while incurring substantial increases in computational cost and noticeable reductions in throughput. By contrast, YOLOv8-n achieves 120 FPS with only 8.1 GFLOPs, representing a favorable accuracy–efficiency trade-off for edge or in-vehicle deployment. It maintains competitive detection performance while preserving sufficient runtime headroom for I/O, visualization, geo-tagging, and downstream decision-making modules. Therefore, we adopt YOLOv8-n as the baseline detector for subsequent ablation studies and for integrating the proposed architectural enhancements.

Under the unified data, training, and evaluation protocol described in Section 2 (fixed train/val/test split, identical optimization and augmentation strategies, single-scale inference at 640 × 640, and a common software/hardware stack), we conducted a systematic baseline comparison of the YOLOv8 family on our road-pothole dataset. Based on this analysis, we select YOLOv8n as the baseline/backbone for the proposed method throughout the remainder of the paper. In terms of accuracy, YOLOv8s and YOLOv8m both achieve an mAP@0.5 of 0.776, providing only a marginal improvement of +0.016 over YOLOv8n (0.760). The larger YOLOv8l and YOLOv8x variants reach 0.772 and 0.771, respectively, without delivering sustained gains. This pattern suggests that, for a single-class detection task at 640 × 640 resolution, the accuracy–per–compute benefit of scaling the network has largely saturated.

By contrast, computational complexity and resource usage grow substantially with model size. FLOPs increase from 8.1 GFLOPs for YOLOv8n to 28.4, 78.7, 164.8, and 257.4 GFLOPs for YOLOv8s/m/l/x, respectively. Parameter counts and model sizes likewise expand from 3.2 M/13 MB (YOLOv8n) to 11.2 M/45 MB (YOLOv8s), 25.9 M/104 MB (YOLOv8m), 43.7 M/175 MB (YOLOv8l), and 68.2 M/273 MB (YOLOv8x). Given the power, thermal, and storage constraints typical of edge- or vehicle-mounted deployments, this combination of limited accuracy gains and sharply increasing cost undermines the practical appeal of the larger variants.

An end-to-end latency analysis further clarifies the performance bottleneck. YOLOv8n exhibits an E2E latency of approximately 8.5 ms per image, YOLOv8s around 10.0 ms, YOLOv8m 21.3 ms, and YOLOv8l and YOLOv8x 38.5 ms and 58.8 ms, respectively. The forward pass dominates the latency budget, and its absolute cost scales roughly in proportion to model size; preprocessing and postprocessing contribute less but also grow for larger models. In operational road-inspection scenarios, available latency headroom governs not only real-time alerting but also the compute allocated to concurrent tasks such as recording, visualization, georegistration, and downstream control. Once E2E latency enters the 20–40 ms range, overall system throughput and stability become significantly constrained.

We therefore adopt a Pareto-frontier perspective in the four-dimensional space of accuracy, compute, latency, and footprint, rather than optimizing a single metric in isolation. Under this criterion, YOLOv8n occupies a favorable cost–performance position: with 8.1 GFLOPs, 3.2 M parameters, a 13 MB model size, and 8.5 ms E2E latency, it achieves an mAP@0.5 of 0.760, which is comparable to that of the larger models. By contrast, the +0.016 mAP gain of YOLOv8s does not compensate for its additional computational load, model size, and latency, and it reduces the compute budget available for robustness-oriented enhancements and modular extensions (e.g., attention, resampling, feature-fusion, or calibration heads). From a maintenance and life-cycle perspective, the smaller footprint of YOLOv8n also facilitates remote distribution and hot upgrades on edge nodes, reducing downtime and operational risk; lower compute and latency further imply reduced average power and thermal stress, which is especially advantageous in enclosed housings and high-temperature environments.

These conclusions are drawn under single-scale 640 × 640 inference on a single device with a unified training protocol. For applications that require higher spatial resolution (e.g., micro-crack or composite-defect detection), increasing the input size (e.g., to 800 × 800) may modestly strengthen the relative advantage of larger models; however, in real-time edge or in-vehicle deployments, the associated increases in latency and power consumption are likely to preserve the overall cost-effectiveness of YOLOv8n. Moreover, our dataset is a single-class, self-compiled road-defect dataset; in multi-class or multi-modal settings, the optimal operating point may shift. To improve external validity, it would be beneficial to report, for example, a seed-reproducibility study or bootstrap confidence intervals to quantify the statistical uncertainty associated with mAP differences on the order of 0.016. From an engineering standpoint, it is also useful to complement the main results with robustness indicators such as mAP or mean performance under common corruptions (illumination changes, fog, blur, compression).

In summary, without compromising engineering feasibility, we select YOLOv8n as the baseline model for this work. The subsequent incremental improvements and ablation studies in Section 3.2 quantify the net gains of lightweight modules such as SPPF-LSKA, DySample, and Coordinate Attention in terms of mAP, end-to-end latency, and GFLOPs. They also provide two practically relevant operating guidelines: (i) the best-performing configuration under a fixed compute budget, and (ii) the achievable accuracy ceiling under a fixed latency target. These results directly support deployment and operational decision-making. If future scenarios substantially relax compute and latency constraints and place a premium on accuracy, YOLOv8s can serve as an upper-bound comparator; however, as summarized in Table 2, its +0.016 mAP improvement does not currently constitute a compelling cost–benefit trade-off given the roughly 3.5× increase in FLOPs, the additional 32 MB of model size, and the higher end-to-end latency.

### 3.2. Comparative Analysis of Ablation Experiments

To validate the effectiveness of the enhancements introduced by the algorithm in this paper, an ablation study was conducted, using the original YOLOv8n network as the baseline. The results of this ablation study are presented in Table 3.

From Table 3, we can see that each module affects the detection behavior in a distinct way. Adding SPPF-LSKA on top of YOLOv8-n primarily improves Recall (+0.025) while also slightly increasing Precision (+0.020) and mAP@0.5 (+0.019). This pattern is consistent with its design: stronger multi-scale context and large-kernel attention help to recover small, blurred, or densely clustered potholes that the baseline tends to miss. DySample, in contrast, yields the largest gain in Precision (+0.047) with only a minor change in Recall (+0.010). This suggests that content-adaptive upsampling mainly stabilizes feature reconstruction in the neck, suppressing false positives caused by noisy background textures and ambiguous edges. When used alone, CA keeps mAP@0.5 at a similar level while slightly reducing parameters and FLOPs (Table 3), acting as a lightweight regularizer that strengthens channel–spatial focus with negligible overhead.

When all three modules are combined in SDC-YOLOv8, their effects become complementary rather than simply additive. SPPF-LSKA first enriches the global context and improves the backbone features for small and irregular defects. DySample then propagates these refined features to higher-resolution maps with less aliasing, which makes the subsequent CA blocks more effective at isolating true pothole regions along the P3/P4/P5 scales. As a result, the full model achieves the highest mAP@0.5 (0.780) and the highest F1 score (0.755) among all variants, with a moderate frame-rate reduction to 85 FPS that remains well within real-time constraints. This interaction analysis clarifies why the joint configuration outperforms any single-module variant and highlights the roles that backbone context modeling, neck-level reconstruction, and head-level attention play in the final detector.

### 3.3. Comparison Experiment of Mainstream Algorithms

Under a unified data split, training strategy, and inference protocol, Table 4 compares six representative detectors in terms of Precision, Recall, mAP@0.5, and FPS. Overall, SDC-YOLOv8 achieves the best performance on mAP@0.5 and Recall—the two metrics most directly linked to task effectiveness—reaching an mAP@0.5 of 0.780 and a Recall of 0.707. Relative to the prevailing lightweight baseline YOLOv8, these correspond to gains of +0.020 in mAP@0.5 and +0.018 in Recall; compared with the strong classical baseline YOLOv5 [33] (mAP@0.5 = 0.770), SDC-YOLOv8 also maintains a clear advantage. Its Precision of 0.810 ranks second, slightly below that of improve1 (0.829); however, improve1 exhibits a notably lower Recall of 0.674, reflecting a conservative operating point with “high precision but low recall”. As a result, its mAP@0.5 (0.766) remains inferior to that of SDC-YOLOv8. Considering the composite F1 score (the harmonic mean of Precision and Recall), SDC-YOLOv8 attains an approximate F1 of 0.755, ranking first among all methods (YOLOv3 [34] ≈ 0.740; YOLOv6 [35] ≈ 0.736; YOLOv5 [36] ≈ 0.729; YOLOv8 ≈ 0.730; improve1 ≈ 0.744). This indicates that, under a fixed deployment threshold, SDC-YOLOv8 achieves the most balanced trade-off between false-positive control and missed-detection suppression.

From the perspective of speed and deployability, the FPS results reveal a clear inverse relationship between model scale and throughput. YOLOv8 (116 FPS), YOLOv5 [36] (111 FPS), and YOLOv6 [35] (110 FPS) form the top tier, fully satisfying high-frame-rate real-time requirements. SDC-YOLOv8 reaches 85 FPS—lower than these three, but still well above the typical real-time threshold of 30 FPS—and thus retains sufficient latency headroom for on-vehicle and edge-node online inspection. In contrast, YOLOv3 achieves only 17 FPS [34], which substantially limits its suitability for real-time deployment and highlights the bandwidth limitations of older, heavier architectures on modern hardware. Viewed jointly across accuracy and efficiency, SDC-YOLOv8 trades moderate additional computation (relative to YOLOv8) for meaningful gains in mAP@0.5 and Recall, representing a favorable engineering cost–benefit profile. By comparison, improve1—although it achieves the highest Precision and is tailored for pavement scenarios [35]—suffers from relatively low Recall, making it less suitable for pothole inspection tasks where missed detections are particularly costly.

Interpreting the relationships among the metrics, mAP@0.5 reflects the average quality of detection and localization, Precision characterizes false-positive control, and Recall quantifies the degree of missed-detection control. Road-pothole inspection is especially sensitive to missed detections, as undetected defects may lead to safety risks and maintenance delays. Under roughly comparable Precision levels across methods, higher Recall therefore contributes more directly to improved operational performance. The higher Recall and mAP@0.5 of SDC-YOLOv8 indicate that it recovers more true potholes and produces tighter bounding boxes in challenging scenes with cluttered backgrounds, low contrast, shadows, and specular reflections. At the same time, its throughput of 85 FPS supports stable video-stream processing and preserves computational headroom for logging, visualization, geo-registration, and downstream control modules. Besides mAP@0.5, we also report mAP@0.5:0.95 (COCO metric) to assess performance under stricter IoU thresholds; SDC-YOLOv8 remains competitive with or superior to YOLOv8s (a larger comparator) and YOLOv5, indicating that its bounding-box localization quality benefits from the proposed modules.

In summary, Table 4 demonstrates that SDC-YOLOv8 achieves joint improvements in accuracy and recall over both traditional and contemporary mainstream detectors, while maintaining an engineering-feasible real-time speed. It leads in the three application-critical dimensions—mAP@0.5, Recall, and F1—and, compared with the fastest models (YOLOv8, YOLOv5, YOLOv6), it deliberately sacrifices part of the frame rate in exchange for more robust detection. This trade-off is well aligned with practical road-defect inspection scenarios, where low miss rates and strong real-time performance are both essential. The quantitative comparison with mainstream detectors is summarized in Table 5.

**Figure 7:**
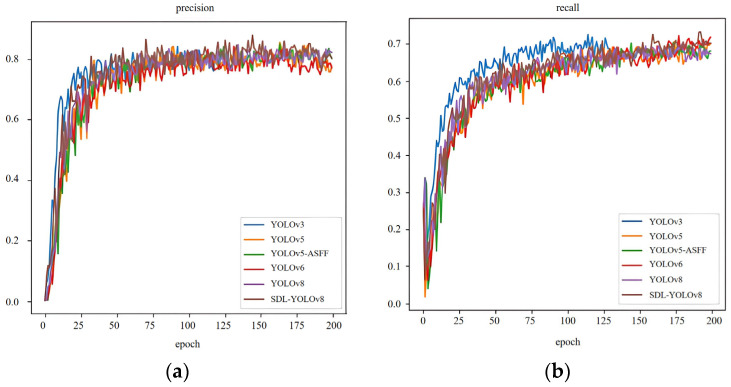

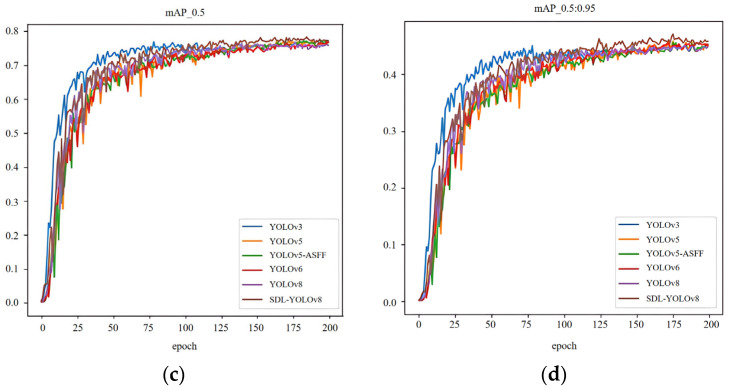
Comparison graphs of metrics for each model: (**a**) Precision curve; (**b**) Recall curve; (**c**) mAP50.5 curve; (**d**) mAP50-95.5 curve. Comparison graph of precision, recall, mAP@0.5, and mAP@0.5–0.95 of each model.

Based on the thorough examination of the experimental results, it is evident that while the detection speed of the refined algorithm presented in this paper has undergone a marginal reduction, its detection accuracy, precision, and recall indices comprehensively outperform other leading detection algorithm models. Notably, the detection accuracy stands at 78%, marking a significant 2 percentage point improvement over the original YOLOv8n algorithm. When considering the computational complexity, detection accuracy, and recall rate of each model, the refined algorithm introduced in this paper emerges as a superior choice, outperforming numerous mainstream detection algorithms. The comparative analysis of the detection results across various indices for each model is illustrated in Figure 7.

As depicted in the figure, the proposed SDC-YOLOv8 model achieves a balanced and superior performance across all key metrics compared to its counterparts. Specifically, it attains the highest precision at 81%, indicating its exceptional ability to minimize false positives—a critical advantage in practical applications where erroneous alerts can undermine system reliability. More importantly, SDC-YOLOv8 also secures the top position in both recall (70.7%) and mAP@0.5 (78%), demonstrating a robust capability to identify a greater number of true potholes while maintaining high localization accuracy. This balanced improvement in precision and recall is particularly noteworthy, as it signifies that the model enhancements effectively mitigate the trade-off that often plagues object detectors, where gains in one metric come at the expense of the other.

While the mAP@0.5–0.95 metric, which evaluates performance under stricter IoU thresholds, presents a more challenging scenario for all models, SDC-YOLOv8 remains highly competitive. Its performance in this regard is on par with or exceeds that of several larger models, such as YOLOv5 and YOLOv8s. This suggests that the introduced modules—SPPF-LSKA, DySample, and CA—collectively enhance the model’s feature representation and localization precision, enabling it to generate higher-quality bounding boxes that align better with the actual defect boundaries. In contrast, while the baseline YOLOv8n and other lightweight models like YOLOv5n achieve high FPS, their lower precision and recall scores highlight a compromise in detection reliability. On the other hand, heavier models do not consistently deliver superior accuracy despite their increased computational costs. The SDC-YOLOv8 model successfully strikes an optimal balance, offering significant gains in detection accuracy without a substantial sacrifice in speed, thereby validating its design as a lightweight yet highly accurate solution for real-world road defect inspection.

### 3.4. Visual Comparison of Detection Results

**Figure 8:**
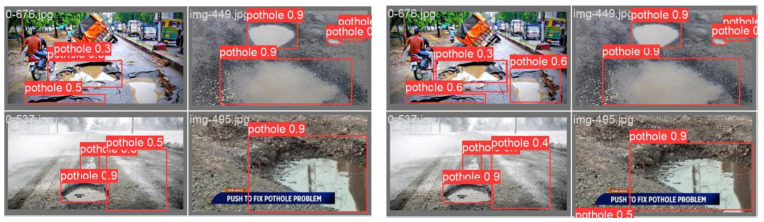
Comparison of visualization results of YOLOv8 and SDC_YOLOv8 on datasets. The (**up**) figure of each comparison chart is YOLOv8, while the (**down**) figure is SDC-YOLOv8.

Figure 8 presents a visual comparison between the original YOLOv8 and the proposed SDC-YOLOv8 across four representative road conditions. Each scene is drawn from a different environment: a cluttered urban roadway with dense, overlapping potholes; a surface with shallow standing water and strong specular reflections; a low-contrast roadway typical of foggy or post-rain conditions; and a large pothole partially occluded by overlaid subtitles in a broadcast frame. The two method panels are aligned in viewpoint and target layout; red boxes denote detections of the class “pothole,” and the numbers indicate the associated confidence scores. By comparing the same locations and objects across the two panels, one can directly assess differences in recall, false-positive control, and localization quality.

In the dense urban scene, SDC-YOLOv8 produces more stable responses for small or boundary-blurred potholes. Instances that receive low confidence under the baseline are assigned higher confidence by the improved model, and the coverage of closely spaced targets is more complete, reducing missed detections. On surfaces with shallow water and strong reflections, the baseline model is prone to misclassifying reflective highlights, water edges, or crack-like textures as potholes. SDC-YOLOv8 suppresses such “pseudo targets” more effectively, maintaining the primary detections while reducing extraneous boxes and yielding a cleaner visual output. Under low-contrast or foggy conditions, the improved model exhibits a more balanced confidence distribution and tighter alignment between predicted boxes and the true pothole boundaries; box positions and scales are less prone to drift, indicating that the enhanced feature representation is more robust under weak texture and low gradient. In the example with subtitle occlusion, SDC-YOLOv8 produces a more compact bounding box around the main pothole and avoids fragmented or duplicate boxes induced by text edges or image noise, improving localization consistency.

Overall, Figure 8 qualitatively demonstrates two salient advantages of SDC-YOLOv8 over the original YOLOv8. First, recall is improved: the enhanced model recovers more true potholes in complex backgrounds, low-contrast scenes, and dense target settings. Second, false-positive control is stronger, particularly in challenging scenarios involving reflections, water surfaces, texture similarity, and partial occlusion. In addition, SDC-YOLOv8 yields more stable confidence scores and tighter geometric alignment of boxes with the underlying defects, reflecting superior environmental robustness and localization quality. These qualitative observations are consistent with the subsequent quantitative results showing accuracy gains with controllable latency overhead, thereby providing intuitive support for the practical value of the proposed method in real-world road inspection. For qualitative comparisons in Figure 8, all visualizations were generated under identical conditions: the same test images, confidence thresholds, and NMS settings were applied to both YOLOv8 and SDC-YOLOv8, ensuring that differences in the displayed bounding boxes solely reflect model behavior.

In practical road-inspection systems, potholes are often detected from continuous video streams rather than from isolated images. In this work, however, all quantitative evaluations are conducted on a curated image dataset derived from smartphone recordings, and the reported throughput (FPS) is measured on single-image inference. Since SDC-YOLOv8 is a one-stage, fully convolutional detector, the same inference pipeline can be directly applied in a frame-by-frame manner to video streams, while the additional computational cost of video decoding and buffering is relatively small on the tested hardware platform. Therefore, the measured FPS provides a reasonable upper bound on the achievable processing rate for real-time video input. A dedicated system-level evaluation on dashcam videos, including end-to-end latency and temporal stability analysis, will be carried out in future work once an appropriate video benchmark and recording platform are available.

## 4. Discussion

### 4.1. Overall Performance and Ablation Summary

This study aimed to identify a detector that jointly balances accuracy, efficiency, and deployability for road–pothole inspection, and to quantify the contributions of three lightweight modules (SPPF-LSKA, DySample, and CA). Under a unified training–evaluation pipeline, the baseline comparison in Table 2 shows that scaling the YOLOv8 backbone beyond the nano variant yields only marginal gains in mAP@0.5, while incurring substantial increases in FLOPs, parameter count, model size, and end-to-end (E2E) latency. Both the quantitative results and qualitative analysis, therefore, support YOLOv8n as a favorable operating point for edge deployment, providing ample latency headroom with only minor accuracy loss.

Building on this choice, the ablation results in Table 3 demonstrate that the proposed SDC-YOLOv8 configuration delivers consistent improvements in Precision, Recall, and mAP over the baseline, with a controlled increase in computational complexity. Compared with YOLOv8n, SDC-YOLOv8 improves mAP@0.5 and Recall while maintaining real-time throughput on the target platform. The comparative study in Table 4 further indicates that SDC-YOLOv8 achieves the highest F1 score among all examined detectors, confirming that the gains are not limited to a single metric but reflect a more favorable balance between false-positive control and missed-detection suppression. We did not include YOLOv9 in our experimental comparison for two practical reasons: (i) at the time of our study, stable YOLOv9 implementations and pre-trained weights optimized for 640 × 640 single-class detection on 8 GB GPUs were not yet widely available; and (ii) our design targets minimal architectural changes on top of YOLOv8n to facilitate integration with existing Ultralytics-based pipelines. Nevertheless, YOLOv9 provides a strong future baseline, and we plan to investigate how SPPF-LSKA, DySample, and Coordinate Attention can complement PGI and task-aligned feature designs in a follow-up study. Under the common training schedule described in Section 2.2, we did not observe obvious divergence or collapse in the loss curves; nevertheless, future revisions will report additional stability indicators (e.g., training/validation curves and seed-wise variation) to make convergence behavior more transparent.

### 4.2. Contributions and Interaction of Lightweight Modules

The ablation study also clarifies how each component contributes to performance. SPPF-LSKA enhances multi-scale aggregation and local selective attention at the deepest backbone stage, leading to higher Recall for densely distributed, small, or partially blurred potholes. DySample replaces fixed interpolation in the neck with sampling-based dynamic upsampling, improving the quality of feature resampling and helping to stabilize bounding boxes in weak-texture regions. The CA module, inserted between the neck and the detection heads, further strengthens representation by emphasizing informative spatial and channel dependencies at modest computational cost. When each module is enabled individually, the ablation results in Table 3 show consistent but complementary gains, indicating that they address different error modes of the baseline detector.

Taken together, these observations suggest that the three modules act on different stages of the pipeline in a mutually reinforcing way: SPPF-LSKA recovers hard positives by enriching deep context, DySample preserves these cues during upsampling and reduces geometric distortion, and CA sharpens the spatial focus of the head and suppresses responses to background clutter. This staged design explains why the full SDC-YOLOv8 configuration achieves the best balance between accuracy and efficiency under the fixed deployment budget. The visual comparisons in Figure 7 and Figure 8 are consistent with this interpretation: relative to the baseline, SDC-YOLOv8 reduces missed detections in cluttered scenes, suppresses false positives caused by specular highlights or shallow water, and produces tighter bounding boxes around defect boundaries. These qualitative improvements align with the quantitative gains in Recall and mAP@0.5.

To our knowledge, prior work has not systematically analyzed the joint effect of deep context enhancement (SPPF-LSKA), sampling-based dynamic upsampling (DySample), and coordinate attention in a unified YOLOv8 framework for road defect detection. The empirical interaction pattern in Table 3—where the full configuration yields a larger improvement in F1 than the sum of individual gains—suggests that these modules reinforce each other across backbone, neck, and head. We view this as an emergent behavior specific to the proposed staged design, rather than a trivial result of isolated module substitution.

### 4.3. Deployment Implications and Robustness

From an operational standpoint, the Pareto relationship between accuracy and efficiency is central. The mAP gap between the nano and larger YOLOv8 variants is small compared with the order-of-magnitude differences in FLOPs and model size, differences that would otherwise restrict concurrent tasks such as recording, visualization, geo-registration, and downstream control on resource-constrained platforms. In the decomposition of E2E latency, the forward pass clearly dominates the runtime budget, and its absolute cost grows approximately in proportion to model complexity, whereas preprocessing and post-processing contribute smaller but non-negligible overheads. Once E2E latency enters the tens-of-milliseconds range, it becomes more difficult to maintain stable throughput and to reserve compute for ancillary tasks.

Within this context, SDC-YOLOv8 deliberately trades a moderate increase in computation for meaningful accuracy gains while staying well above typical real-time thresholds. Because the proposed modules are lightweight and operate on progressively downsampled feature maps, they preserve sufficient headroom for system-level optimizations such as kernel fusion, TensorRT or ONNX deployment, mixed-precision or INT8 quantization, and structured pruning. These techniques can further reduce wall-clock latency and power consumption without requiring architectural changes. In addition, the unified training and inference protocol—with fixed input resolution, batch size, thresholds, and hardware/software stack—provides a reproducible baseline for future replication and extension. In a subsequent revision, we plan to complement the reported point estimates with variance analyses across random seeds and bootstrap-based confidence intervals, thereby strengthening the statistical robustness of the observed improvements.

In terms of cost, SDC-YOLOv8 sacrifices about 35 FPS of throughput compared with YOLOv8n on the same hardware (from 120 FPS down to 85 FPS), corresponding to an E2E latency increase from 8.5 ms to roughly 11.8 ms per frame. The parameter count and model size increase from 3.2 M/13 MB to 3.36 M/14 MB. For most road-inspection applications, this trade-off is acceptable, but in ultra-high-frame-rate or multi-task systems (e.g., joint detection and tracking on low-power SoCs), the additional latency may require further compression and hardware-specific optimization.

Finally, we emphasize that our claim of deployability is currently based on image-level evaluation and single-device profiling, rather than a fully instrumented on-vehicle deployment. All throughput measurements are obtained on a desktop-class GPU (TITAN X) with single-image inference; embedded platforms such as Jetson-class devices may exhibit different absolute latencies and power characteristics. Moreover, we do not yet include dedicated nighttime, heavy rain, or thick fog subsets, nor do we evaluate temporal behavior on continuous driving videos. These aspects will be addressed in future work by (i) collecting and annotating a video benchmark spanning diverse weather and lighting conditions, and (ii) porting SDC-YOLOv8 to representative embedded SoCs to measure end-to-end performance under realistic bandwidth and power constraints.

### 4.4. Limitations and Future Work

Several limitations should also be acknowledged. First, the dataset is single-class and relatively small compared with the variability of real-world roads. Domain shifts may occur across pavement materials, camera types, mounting positions, weather, and illumination. Although the experiments include challenging scenes, the robustness analysis is necessarily incomplete; performance under severe fog, heavy rain, nighttime glare, strong compression, or extreme motion blur remains to be systematically assessed. Extending the dataset across regions, seasons, and sensors—and using active learning to prioritize hard negatives such as puddles, tar patches, and shadows—is expected to improve generalization and reduce failure modes.

Second, all latency and throughput measurements were collected on a single hardware platform. While FLOPs offer device-agnostic comparability, profiling on multiple devices (e.g., embedded GPUs, automotive SoCs, and low-power edge CPUs) would strengthen the external validity of the deployment analysis and help map SDC-YOLOv8 to specific application scenarios and hardware budgets. Third, the reported mAP and related metrics are currently given as point estimates. For improvements on the order of a few percentage points, uncertainty quantification becomes important: future work will incorporate seed-repeated runs, variance analysis, and paired significance tests or bootstrap-based confidence intervals to better characterize the reliability of the reported gains and the stability of training.

These limitations indicate several concrete directions for future research. On the data side, we will expand coverage across diverse road types and environmental conditions, and explore semi-supervised or weakly supervised learning to reduce annotation cost. On the model side, incorporating temporal cues (e.g., short-term tracking, temporal filtering, or video-based feature aggregation) may stabilize detections in bumpy or jittery video, while multi-task learning (e.g., joint detection and segmentation or surface-type classification) could reduce false alarms in texture-ambiguous scenarios. On the deployment side, structured compression (quantization-aware training, low-rank factorization, and channel pruning) and runtime optimizations (TensorRT/ONNX deployment, asynchronous I/O, and batched NMS) can further shorten E2E latency and lower power consumption. Finally, integrating reliability measures—such as confidence calibration (ECE/Brier score), abstention policies, and model health monitoring—would support threshold selection, facilitate alarm management, and enhance safe operation in long-term production environments.

Despite these gains, SDC-YOLOv8 still fails in certain edge cases. For example, severe motion blur, near-complete occlusion by vehicles or pedestrians, and extreme over- or under-exposure can cause both the baseline and the improved model to miss potholes or to confuse dark shadows with defects. In dense urban scenes with complex markings, small manhole covers and patchwork repairs occasionally produce false positives. A more detailed failure-case taxonomy and targeted augmentation strategies are part of our ongoing work. It is worth noting that the public dataset used in this study does not provide formal metadata describing whether each image was captured in a “city-center” or “suburban” area. Because of this limitation, we did not conduct an additional quantitative ablation study on strictly defined city-center versus suburban subsets in this work. Instead, we qualitatively verified that the test split includes both dense urban streets and more open suburban or rural roads, including scenes with strong specular reflections, water puddles, and low-contrast damage (see Figure 8). In future work, we plan to extend our dataset with explicit geo-spatial tags and fine-grained scene-level annotations (e.g., city center, suburban, rural), which will enable a more systematic comparison of SDC-YOLOv8 across different deployment environments.

Nevertheless, the single-class and limited-size nature of the dataset implies that domain shift remains a concern: variations in lighting (nighttime, backlighting), weather (heavy rain, fog, snow), camera optics, mounting height, and regional pavement types may degrade performance when deploying the model beyond the collected scenes. Our current analysis only partially covers these factors; a more systematic robustness evaluation across cities, seasons, and sensor setups is left to future work. Beyond single-class pothole detection, the proposed design is readily extensible to multi-class pavement defects (e.g., cracks, patches, rutting) by adding class labels and re-training the detection heads. The error-pattern–driven module placement (deep context for small/irregular targets, dynamic upsampling for texture-rich surfaces, coordinate attention for localization) is not specific to roads and could be transferred to other infrastructure and industrial inspection tasks, such as bridge surface assessment or printed-circuit-board defect detection. Incorporating temporal information from video—for instance, via short-term feature aggregation or lightweight tracking—represents another promising direction to further stabilize detections under camera shake and vehicle motion.

## 5. Conclusions

Road surface defect detection remains a critical challenge for intelligent transportation systems, requiring detectors that balance accuracy, efficiency, and deployability on resource-constrained platforms. This study addresses these requirements through SDC-YOLOv8, a lightweight detection model that enhances YOLOv8 via attention-enhanced feature learning and adaptive feature reconstruction. Specifically, the model integrates three complementary technical innovations—SPPF-LSKA for multi-scale feature representation, DySample for adaptive feature reconstruction, and Coordinate Attention for robust localization—into the YOLOv8 architecture.

Systematic evaluation on a public pothole dataset demonstrates that SDC-YOLOv8 achieves an mAP@0.5 of 0.780, Precision of 0.810, and Recall of 0.707 at 85 FPS. Compared to the YOLOv8n baseline, the proposed method improves mAP@0.5 by 2.0 percentage points and Recall by 1.8 percentage points, yielding an F1 score of 0.755 that surpasses YOLOv3/5/6/8 and their improved variants. Ablation studies confirm that each module contributes complementary benefits: SPPF-LSKA strengthens irregular target modeling, DySample enhances detail reconstruction at modest cost, and Coordinate Attention improves localization precision. Qualitative analysis further reveals stronger false-positive suppression and missed-detection control in challenging conditions, including specular reflections, low contrast, and dense small objects.

From a deployment perspective, SDC-YOLOv8 strikes a favorable balance between accuracy and computational efficiency, operating within real-time constraints while maintaining sufficient headroom for concurrent tasks such as video streaming, visualization, and decision-making. Its lightweight architecture (3.5 M parameters, 14 MB model size) facilitates edge deployment and enables rapid distribution and updates in production environments. These results demonstrate that targeted architectural refinements can yield substantial performance gains without compromising real-time capability, offering a practical and field-ready solution for intelligent road inspection and infrastructure maintenance systems. The present study focuses on static-image evaluation, and extending SDC-YOLOv8 to a fully tested video-based inspection system will be an important direction of our future work.

## Figures and Tables

**Table 1:** Training parameter configuration.

Parameter	Configure	Parameter	Configure
Epochs	200	Optimizer	SGD
Batch size	8	Close_Mosaic	10
Workers	4	Momentum	0.937
Imgsz	640	Ir0	0.01

**Table 2:** Configuration and overhead of the proposed modules in SDC-YOLOv8 (relative to YOLOv8-n).

Module	Inserted Stage	Key Settings	Δ Params	Δ GFLOPs	Description
SPPF-LSKA	Backbone, P5 output	k = 21 (factorized), d = 3, r = 4	+0.08 M	+0.2	Replaces SPPF; enhances multi-scale context and long-range dependencies.
DySample	Neck, P5→P4 and P4→P3 paths	scale factor = 2, 4 sampling points	+0.05 M	+0.3	Replaces nearest upsampling; improves detail reconstruction.
CA	Before heads on P3/P4/P5	reduction ratio r = 16	+0.03 M	+0.1	Adds direction-aware and position-aware attention for localization.
Total	–	–	+0.16 M	+0.6	Overall overhead of SDC-YOLOv8 vs. YOLOv8-n.

**Table 3:** Benchmark algorithm prior experiment.

Model	mAP@0.5	GFLOPs	Params (M)	Model Size (MB)	E2E Latency (ms)
YOLOv8n	0.760	8.1	3.2	13	8.5 (1.0/6.8/0.7)
YOLOv8s	0.776	28.4	11.2	45	10.0 (1.2/7.8/1.0)
YOLOv8m	0.776	78.7	25.9	104	21.3 (2.0/18.0/1.3)
YOLOv8l	0.772	164.8	43.7	175	38.5 (3.0/34.0/1.5)
YOLOv8x	0.771	257.4	68.2	273	58.8 (4.0/53.0/1.8)

**Table 4:** Ablation results on the self-compiled pothole test set. All metrics are reported on the held-out test subset.

Models	mAP@0.5	Prediction	Recall	F1	FPS
YOLOv8	0.760	0.777	0.689	0.730	120
+SPPF_LSKA	0.779	0.797	0.714	0.753	117
+DySample	0.773	0.824	0.699	0.756	106
+CA	0.773	0.815	0.693	0.749	104
Ours	0.780	0.810	0.707	0.755	85

**Table 5:** Comparison of performance indexes of mainstream high-precision target detectors (test set).

Models	Precision	mAP@0.5	Recall	F1	FPS
YOLOv3 [34]	0.787	0.768	0.698	0.740	17
YOLOv5 [36]	0.779	0.770	0.685	0.729	111
YOLOv6 [35,37]	0.77	0.763	0.704	0.736	110
improve1	0.829	0.766	0.674	0.744	93
YOLOv8	0.777	0.760	0.689	0.730	116
Ours	0.810	0.780	0.707	0.755	85

## Data Availability

The original contributions presented in the study are included in the article. Further inquiries can be directed to the corresponding author.
